# Oral Kaposi Sarcoma in two patients living with HIV despite sustained viral suppression: New clues

**DOI:** 10.4317/jced.59610

**Published:** 2022-05-01

**Authors:** Clement Messeca, Matthieu Balanger, Florent Geoffroy, Xavier Duval, Mahtab Samimi, Sarah Millot

**Affiliations:** 1 DDS, Odontology department, Bichat Hospital, Paris, France.Faculty of dentistry; 2 DDS, Oral surgery, Paris, France; 3 DDS, Oral surgery department, Department of oral surgery Montpellier Hospital CHRU, France. Faculty of dentistry; 4 MD, PhD, Titular professor, Center of Clinical Investigations, department of infectious diseases. INSERM 1425, Bichat Hospital, AP-HP, Paris-Diderot University, Inserm U1137, Paris, France; 5 MD, PhD, Titular professor, Dermatology department, Tours Hospital CHU, University of Tours, 37000 Tours; 6 DDS, PhD, Associate professor, Montpellier Cancer Institute (ICM), University of Montpellier, Montpellier, France

## Abstract

Kaposi sarcoma (KS) etiologically linked to Kaposi sarcoma-associated herpesvirus (KSHV) is the most common HIV associated cancer despite the generalization of antiretroviral therapy. Head, neck, and especially oral cavity are common and specific sites for lesions. Those oral lesions contain a high viral load of KSHV virus and are one of the signs of disease severity. The development of KS in HIV-infected patients is classically described in case of low CD4 count, but recently reported cases revealed oral KS despite a robust CD4 count, such observations being possibly linked to interactions between periodontal germs and the oncogenic KSHV virus. We present two cases of KS location on the oral mucosa in HIV patients with gingival inflammation despite efficient antiretroviral treatment and immune restoration. Those cases suggest that the diagnosis of oral KS lesions should be considered by oral surgeons, dermatologists, and infectious specialists, when managing any suspicious lesion in an HIV patient, even with undetectable HIV viral loads, or in a seronegative patient with unprotected sexual activity.

** Key words:**Kaposi Sarcoma, KSHV, HIV infection, CD4 count, oral lesion, oral cancer, periodontal pathogens.

## Introduction

Kaposi Sarcoma Associated Herpes Virus (KSHV) is the etiologic agent of Kaposi sarcoma (KS) a neoplasm of endothelial origin (1). The four clinical-epidemiological types of KS are classic, endemic, HIV associated, and transplant associated KS. In HIV negative patients, KS lesions predominantly manifest in the skin (lower extremities) and lymph node, while oral location is extremely rare (2).

In contrast, in HIV-related KS, lesions in the oral cavity (oral mucosa) are typical and observed in up to 60% to 80% of patients (3). Despite a significant decrease in the incidence of KS due to the spread of antiretroviral therapy, it still remains the most common malignancy in HIV patients causing significant morbidity and mortality (4). One of the main factors recognized to promote the development of oral lesions in HIV patients has long been alteration of immune system especially of CD4 cells. However recent publications describe forms of KS in HIV-positive patients with high CD4 counts and undetecTable viral load which opens up avenues of reflection for these new entities(5-7). Moreover, it has been shown that in HIV patients, KSHV is detecTable in saliva across all levels of CD4 counts (8). KSHV has tropism for oral epithelial cells, is transmitted through saliva and presents significantly high seroprevalence in individuals with high-risk sexual behavior and in HIV positive patients (9). Oral KS lesions present higher virus load than cutaneous lesions(10). Given the fact that this cancer may persist in oral cavity despite a robust CD4 level, other factors affecting oral KS occurrence have been suggested. Recent publications highlight that interactions between KSHV and the oral microbiome (especially periodontal pathogens) may reactivate the virus from latency in co infected KSHV/ HIV patients(11-12). On the other hand, it has been shown that gingival inflammation and periodontal disease are significantly increased in HIV patients. This supports the hypothesis of a link between oncogenic viruses and periodontal bacteria that may facilitate the progression of oral KS in HIV patients (13). These hypotheses suggest that the diagnosis of oral KS should be considered by oral surgeons, dermatologists, infectious specialists when managing any suspicious lesion in an HIV patient even with high CD4 count or even in a seronegative patient with unprotected sexual activity (14).

Here, we present two clinical cases demonstrating oral lesions of KS in patients living with HIV who were virologically controlled and with high levels of CD4.

## Case Report

**Figure 1:**
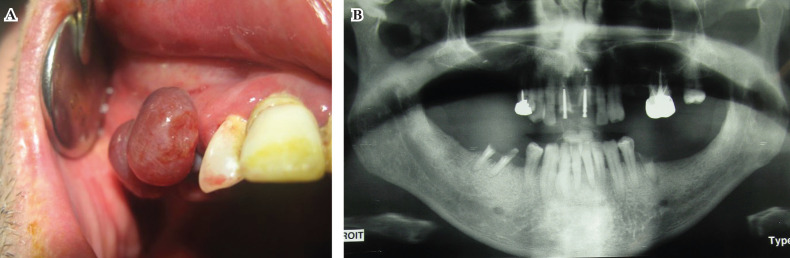
Clinical view of the violaceous, exophytic mass of the right maxilla oral mucosa mass (A), Panoramic radiograph for evaluation of the presence of infectious dental apical lesions (B).

**Figure 2:**
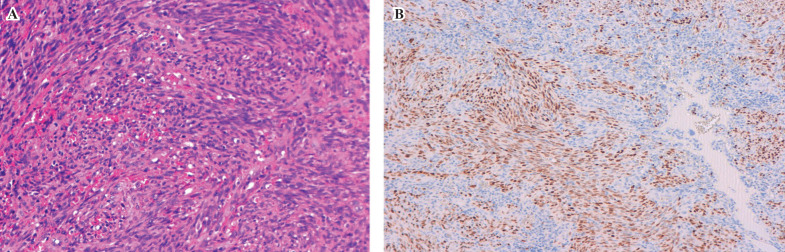
Histopathological analysis confirmed the diagnosis of Kaposi’s sarcoma with proliferation of spindle cells (A) and positive immunostaining with an antibody to HHV8 (X 20 magnification) (B).

-Patient 1:

A 56-year-old Caucasian man was referred in our department for a maxillary intra oral nodule that had progressed for two weeks. He had initially consulted an oral surgeon with concern for tooth infectious foci. This patient had been diagnosed with HIV eight years before and was treated with stabilized anti-retroviral therapy (darunavir, andemtricitabine). Laboratory results revealed CD4 count of 803/mm3 and a plasmatic HIV viral load below 20 copies/ml. At the intra oral examination, we observed a soft purplish round gingival pedunculated mass of the right maxilla extending from the canina to the 2nd premolar site (Fig. 1A). The lesion rapidly enlarged and became exophytic. We observed non tender gum inflammation and poor oral hygiene. An intra oral radiography (Fig. 1B) ruled out apical lesions related to the teeth but revealed exposed tooth root in the mandible. No further oral mucosal or skin lesions could be observed. An excisional biopsy under local anesthesia was performed and histopathological examination confirmed the diagnosis of Kaposi sarcoma (Fig. 2). An extensive assessment (thoracic and abdominal computed tomography) revealed involvement of cervical lymph nodes, and the patient was discharged in infectious disease department.

-Patient 2.

A 47-year-old African woman man was referred for a mass on tongue of 6-month duration. She had been diagnosed with HIV-1 infection 20 years before. She had remained clinically sTable with antiretroviral therapy (maraviroc, tenofofir disoproxil fumarate). Her past medical history included obesity, hypertension, and obstructive sleep apnea.

**Figure 3:**
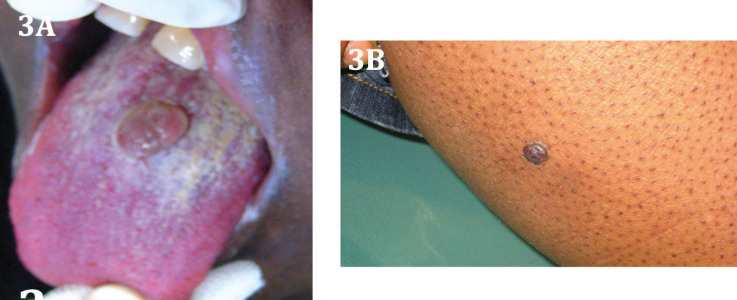
Clinical view of the oral lesion, a nodule of dorsum of the tongue (A) and the brown cutaneous papule on the leg (B).

At the day of the consultation, CD4 lymphocyte count was 754 cells/mm3 and plasmatic HIV-1 viral load was below 20 copies/ml. At intra oral examination, she originally presented a red, elastic and soft nodule, in the center of the dorsal tongue (Fig. 3A) and gingival inflammation. Physical examination revealed a similar lesion in leg (Fig. 3B). A excisional biopsy of the lesion on the tongue showed a spindle cell neoplasm positive on immunohistochemical staining for HHV8, confirming the diagnosis of KS.

A chest computed tomography did not reveal any other location of KS. The patient was referred to the Infectious department for the introduction of systemic chemotherapy by pegylated liposomal doxorubicin.

## Discussion

These two clinical cases demonstrate oral KS lesions in patients living with HIV who were virologically controlled and with high levels of CD4; of note, these two patients had poor oral hygiene with gingival inflammation. The atypical context of the immunovirological status of these patients probably led to a diagnostic delay. In the case of one of the two patients, KS lesion had lasted for more than 6 months before a specific consultation was requested. In these two cases, KS was associated either with a cutaneous lesion or a lymph node. It’s highlights the need for this extensive assessment because the disease is more aggressive with frequent disseminated lesions.

Lesions of the oral mucosa in KS are very specific of the HIV associated KS type. In the other clinical forms of KS, manifestations in the mouth are extremely rare, (18). Case reports have highlighted oral Kaposi lesions in the context of patients effectively treated for their HIV infection and in patients without HIV but with unprotected homosexual behavior (17-18). Moreover, KSHV infection has a very high prevalence in those patients.

It has been shown very recently that HIV / KSHV co-infected individuals harbor changes in their oral microbiota; and periodontopathogens, specifically *Staphylococcus aureus* are able to reactivate a KSHVs latent phase and thus the progression of KS (11). Periodontitis, frequent in HIV patients, is involved in a wide range of diseases and may thus have a role in KS. This hypothesis could alternatively explain the evolution of oral KS in patients positive for HHV8, but without HIV infection. Furthermore, interactions between oncogenic viruses and oral bacteria could be an explanation for KS persistence despite high CD4 counts in HIV patients.

## Conclusions

This evolution in the pattern of oral lesions means that KS should be considered in (i) all patients living with HIV regardless of their immune status and CD4 count, and (ii) in patients with risky sexual behaviors (population in which KSHV transmission is significant). In a very practical way, any persistent dark or purlish nodule especially on palate, tongue or gingiva must be biopsied and raise suspicion of underlying HIV infection. Dermatologists, infectious specialists and dentists should be familiar with the various clinical manifestations of KS and must keep in mind that the oral involvement of a KS is typically – but not constantly- related to the HIV-associated form of KS. Ours patient’s case challenges the common perception ok KS as an HIV defining illness in patients with low CD4 counts.
